# Supplementary material for: MicroRNAs in Small Extracellular Vesicles Indicate Successful Embryo Implantation during Early Pregnancy
Source: Cells. 2020 Mar 6;9(3):645. doi: 10.3390/cells9030645 (PMC7140406; doi:10.3390/cells9030645)
Supplement: Supplementary file 1 [file cells-09-00645-s001.zip › Supplementary material/Supplementary Figures.docx]

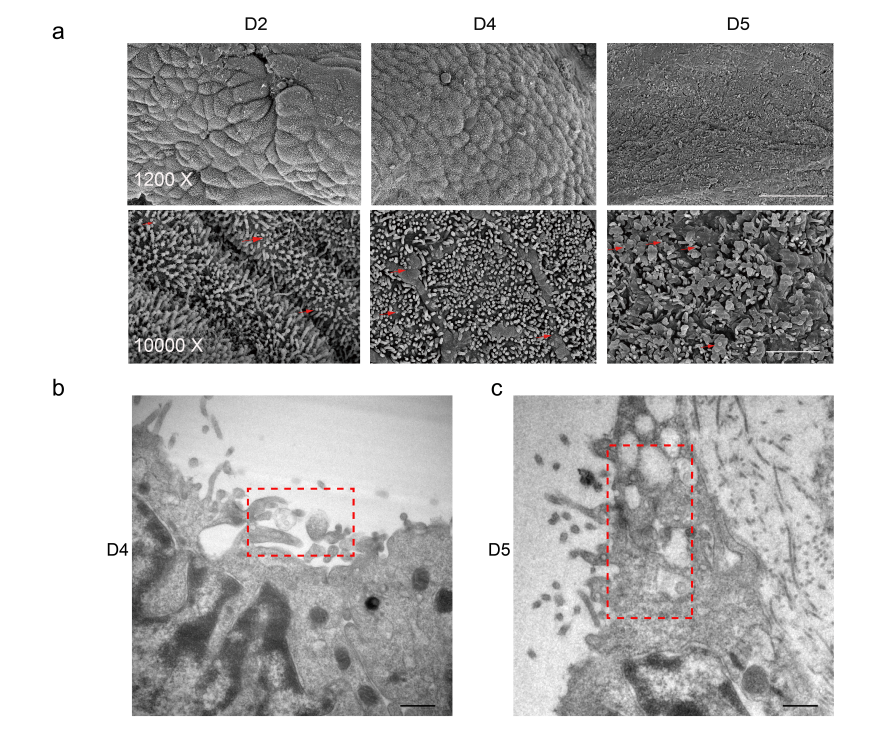


Figure S1. Small extracellular vesicles (sEVs) were secreted by endometrium during implantation. (a) SEM images shown the morphology of mouse endometrial surface on D2, D4, and D5 of pregnancy. Red arrows show the vesicles on the surface of endometrial epithelium similar to sEVs. Bar, 1200×: 30 μm, 10,000×: 3 μm. Electron microscopic images of sEVs in endometrium on D4 (b) and D5 (c). Red box indicated sEVs.
